# Clinical illness and outcomes in Nigerian children with persistent early-appearing anaemia following initiation of artemisinin-based combination treatments of uncomplicated falciparum malaria

**DOI:** 10.1051/parasite/2019058

**Published:** 2019-09-13

**Authors:** Kazeem Akano, Bayo Fatunmbi, Godwin Ntadom, Adejumoke I. Ayede, Temitope Aderoyeje, Adewale Bakre, Omobolaji T. Alebiosu, Odafe Akpoborie, Chukwuebuka Okafor, Grace O. Gbotosho, Onikepe A. Folarin, Joy C. Ebenebe, Jose Ambe, Robinson Wammanda, Nma Jiya, Finomo Finomo, George Emechebe, Olugbenga Mokuolu, Chimere Agomo, Stephen Oguche, Christian Happi, Akintunde Sowunmi

**Affiliations:** 1 Antimalarial Therapeutic Efficacy Monitoring Group, National Malaria Elimination Programme, The Federal Ministry of Health Abuja 900211 Nigeria; 2 Department of Biological Sciences and African Centre of Excellence for Genomics of Infectious Diseases (ACEGID), Redeemer’s University Ede 232102 Nigeria; 3 Institute for Medical Research and Training, College of Medicine, University of Ibadan Ibadan 200212 Nigeria; 4 World Health Organization, Country Office Kampala Uganda; 5 Department of Pharmacology and Therapeutics, College of Medicine, University of Ibadan Ibadan 200284 Nigeria; 6 Department of Paediatrics, University of Ibadan Ibadan 200284 Nigeria; 7 Department of Clinical Pharmacology, University College Hospital Ibadan 200212 Nigeria; 8 Department of Pharmacology and Toxicology, Faculty of Pharmacy, University of Ibadan Ibadan 200284 Nigeria; 9 Department of Paediatrics, Nnamdi Azikiwe University Awka 420110 Nigeria; 10 Department of Paediatrics, University of Maiduguri Maiduguri 600230 Nigeria; 11 Department of Paediatrics, Ahmadu Bello University Zaria 810001 Nigeria; 12 Department of Paediatrics, Usman Dan Fodio University Sokoto 840001 Nigeria; 13 Department of Paediatrics, Federal Medical Centre Yenagoa 560231 Nigeria; 14 Department of Paediatrics, Imo State University Teaching Hospital Orlu 473212 Nigeria; 15 Department of Paediatrics and Child Health, University of Ilorin Ilorin 240003 Nigeria; 16 Department of Medical Laboratory Science, University of Lagos Lagos 100254 Nigeria; 17 Department of Paediatrics, University of Jos Jos 930222 Nigeria

**Keywords:** Persistent early-appearing anaemia, Falciparum malaria, Artemisinin-based combination treatments, Children, Nigeria

## Abstract

In non-anaemic children with malaria, early-appearing anaemia (EAA) is common following artemisinin-based combination treatments (ACTs) and it may become persistent (PEAA). The factors contributing to and kinetics of resolution of the deficit in haematocrit from baseline (DIHFB) characteristic of ACTs-related PEAA were evaluated in 540 consecutive children with malaria treated with artemether-lumefantrine, artesunate-amodiaquine or dihydroartemisinin-piperaquine. Asymptomatic PEAA occurred in 62 children. In a multiple logistic regression model, a duration of illness ≤3 days before presentation, haematocrit <35% before and <25% one day after treatment initiation, drug attributable fall in haematocrit ≥6%, and treatment with dihydroartemisinin-piperaquine independently predicted PEAA. Overall, mean DIHFB was 5.7% (95% CI 4.8–6.6) 7 days after treatment initiation and was similar for all treatments. Time to 90% reduction in DIHFB was significantly longer in artemether-lumefantrine-treated children compared with other treatments. In a one compartment model, declines in DIHFB were monoexponential with overall mean estimated half-time of 3.9 days (95% CI 2.6–5.1), Cmax of 7.6% (95% CI 6.7–8.4), and Vd of 0.17 L/kg (95% CI 0.04–0.95). In Bland-Altman analyses, overall mean anaemia recovery time (AnRT) of 17.4 days (95% CI 15.5–19.4) showed insignificant bias with 4, 5 or 6 multiples of half-time of DIHFB. Ten children after recovery from PEAA progressed to late-appearing anaemia (LAA). Progression was associated with female gender and artesunate-amodiaquine treatment. Asymptomatic PEAA is common following ACTs. PEAA or its progression to LAA may have implications for case and community management of anaemia and for anaemia control efforts in sub-Saharan Africa where ACTs have become first-line antimalarials.

**Trial registration:** Pan Africa Clinical Trial Registration PACTR201709002064150, 1 March 2017 http://www.pactr.org

## Abstract (fr)

Chez les enfants impaludés non anémiques, une anémie d’apparition précoce (AAP) est fréquente après un traitement par une combinaison à base d’artémisinine (CTA) et peut devenir persistante (AAPP). Les facteurs contribuant à la résolution du déficit en hématocrite par rapport aux valeurs initiales (DHPVI), caractéristique de l’AAPP liée aux CTA, ont été évalués chez 540 enfants impaludés traités par artéméther-luméfantrine, artésunate-amodiaquine ou dihydroartémisinine-pipéraquine. Une AAPP asymptomatique est survenue chez 62 enfants. Dans un modèle de régression logistique multiple, une durée de la maladie ≤ 3 jours avant la présentation, un hématocrite < 35 % avant et < 25 % un jour après le début du traitement, une chute attribuable au médicament ≥ 6 % et un traitement par dihydroartémisinine-pipéraquine prédisent l’AAPP de manière indépendante. Globalement, le DHPVI moyen était de 5,7 % (IC à 95 % de 4,8 à 6,6) 7 jours après le début du traitement et était similaire pour tous les traitements. Le temps de réduction de 90 % du DHPVI était significativement plus long chez les enfants traités à l’artéméther-luméfantrine que pour les autres traitements. Dans un modèle à un compartiment, les baisses de DHPVI étaient monoexponentielles avec une demi-durée moyenne globale estimée à 3,9 jours (IC 95 % 2,6–5,1), une Cmax de 7,6 % (IC 95 % 6,7–8,4), et une Vd de 0,17 L/kg. (IC 95 % 0,04–0,95). Dans les analyses de Bland-Altman, le temps moyen de récupération de l’anémie totale de 17,4 jours (IC à 95 % de 15,5 à 19,4) a montré un biais non significatif avec 4, 5 ou 6 multiples de la moitié de la demi-vie du DHPVI. Dix enfants après convalescence de AAPP ont évolué vers une anémie tardive. La progression était associée au sexe féminin et au traitement par l’artésunate-amodiaquine. L’AAPP asymptomatique est courante après les CTA. L’AAPP ou sa progression vers l’anémie tardive peut avoir des implications pour la gestion individuelle ou communautaire des cas d’anémie et pour les efforts de contrôle de l’anémie en Afrique subsaharienne où les CTA sont devenus des antipaludéens de première ligne.

**Abbreviations**
%: Percent
°C: Degree Celsius
AA: Artesunate-amodiaquine
ACPR: Adequate clinical and parasitological response
ACTs: Artemisinin-based combination treatments
AL: Artemether-lumefantrine
ANOVA: Analysis of variance
AOR: Adjusted odds ratio
AUC_dihfb_: Area under the curve of the deficit in haematocrit from baseline *versus* time
AnRT: Anaemia recovery time
CI: Confidence interval
CLp_dihfb_: Plasma clearance of the deficit in haematocrit from baseline
Cmax_dihfb_: Maximum deficit in haematocrit from baseline
DAFH: Drug-attributable fall in haematocrit
DHP: Dihydroartemisinin-piperaquine
DIHFB: Deficit in haematocrit from baseline
dL: Deciliter
EAA: Early-appearing anaemia
FCT: Fever clearance time
g: Gram
GMPD: Geometric mean parasite density
Hb: Haemoglobin
HCT: Haematocrit
*K*_eldihfb_: Elimination rate constant of deficit in haematocrit from baseline
*K*_eldiHbfb_: Elimination rate constant of deficit in haemoglobin from baseline
kg: Kilogram
L: Litre
LAA: Late-appearing anaemia
OR: Odds ratio
PADH: Post-artesunate delayed haemolysis
PCR: Polymerase chain reaction
PCT: Parasite clearance time
PEAA: Persistent early-appearing anaemia
PRR: Parasite reduction ratio
*t*_1/2dihfb_: Half-time of deficit in haematocrit from baseline
*t*_1/2diHbfb_: Half-time of deficit in haemoglobin from baseline
*T*_50dihfb_: Time to 50% reduction of deficit in haematocrit from baseline
*T*_90dihfb_: Time to 90% reduction of deficit in haematocrit from baseline
Tmax_dihfb_: Time to reach maximum deficit in haematocrit from baseline
Vd_diHbfb_: Volume of the distribution of the deficit in haemoglobin from baseline
μL: Microliter

## Introduction

Falciparum malarial anaemia, one of the inevitable consequences of untreated and treated infections, is a public health problem in many malaria endemic areas of the world [6, 11, 12, 14, 18, 21, 22]. Although intravenous artesunate or artemisinin-based combination treatments (ACTs) have remained efficacious treatments for severe or uncomplicated falciparum malaria, treatment is associated with delayed haemolytic anaemia (post-artesunate delayed haemolysis [PADH] syndrome) in severe malaria in immunologically naïve adults [3, 9, 10, 16, 28, 30], or a relatively asymptomatic late-appearing anaemia (LAA) in uncomplicated infections in children [19]. Treatment may also be associated with an early-appearing anaemia (EAA) in children who are not anaemic before treatment initiation [19] and it may persist for up to one or more weeks after treatment initiation – persistent early-appearing anaemia (PEAA). Recovery from PEAA is common but it can progress after recovery to LAA [21].

ACT-related PEAA has not been broadly evaluated clinically or parasitologically, and its relationship to LAA has not been frequently explored in young African children. Additionally, little is known about the time-course of the deficit in haematocrit characteristic of ACT-related PEAA. Clinical and parasitological evaluation, and evaluation of the time-course of PEAA may assist in the management of individual patients and contribute to community management of malaria- and antimalarial-related anaemia in young African children.

In a previous study, we described the clinical illness and outcomes in children with LAA following ACTs of uncomplicated falciparum malaria [19]. In the present study, we describe the clinical illness and outcomes in Nigerian children with PEAA following initiation of ACTs of uncomplicated falciparum malaria. The main aims of our study were: to determine the frequency, and the factors contributing to PEAA and to evaluate the time-course of the deficit in haematocrit characteristic of ACT-related PEAA following initiation of treatment with artemether-lumefantrine (AL), artesunate-amodiaquine (AA) or dihydroartemisinin-piperaquine (DHP). Additional aims were to determine the relationship between anaemia recovery time (AnRT) and the half-time of the deficit in haematocrit in children with ACT-related PEAA, and the time-course of progression from PEAA to LAA.

## Patients and methods

### Study design

The study took place between June 2014 and September 2015. It was part of a larger study to monitor therapeutic efficacies of AL, AA and DHP in <5-year-old children with malaria living in six geographical areas of Nigeria. The details of the therapeutic efficacies have been reported elsewhere [7]. The present study is a consecutive study of all <5-year-old children with uncomplicated falciparum malaria who satisfied the criteria for the definition of persistent early appearing anaemia (PEAA) following treatment with ACTs (see below).

### Study procedures

Patients were eligible to participate in the study if they were aged 6–59 months, had symptoms compatible with acute uncomplicated malaria and *Plasmodium falciparum* mono-infections between 2000 and 200,000 μL^−1^ of blood, had no history of antimalarial drug intake in the 2 weeks preceding enrolment, and no evidence of severe malaria [25, 29], and parents or guardians gave written informed consent. Patients were randomised to AL, AA or DHP treatments for 3 days (day 0–2), as previously described [7]. The day of presentation (day of treatment initiation) was regarded as day 0. Thick and thin blood films were obtained from each child as soon as they came to the clinic and the slides were carefully labelled with the patients’ codes and air-dried before being Giemsa-stained. Routine clinical and parasitological evaluations were done at enrolment and during follow-up on days 1–3 or 1–4, 7, 14, 21, 28, 35 and 42, as previously described [7].

### Haematological evaluation

Capillary blood obtained from a finger prick was collected before treatment and during follow-up, and was used to measure haematocrit using a microhaematocrit tube and microcentrifuge (Hawksley, Lancing, UK). Anaemia was defined as haematocrit <30% and was further classified as mild, moderate or severe if haematocrit was 21–29, 15–<21 or <15% [14, 21]. LAA was diagnosed as previously described using the following criteria: adequate clinical and parasitological response (ACPR) [26] occurring within 1 week, haematocrit ≥30% at 1 and/or 2 weeks, a fall in haematocrit to <30% occurring at 3–6 weeks, absence of concomitant illness at 1–6 weeks, and absence of asexual parasitaemia by both microscopy and PCR at 1–6 weeks [19]. In patients who had PEAA or LAA, AnRT was defined as time from appearance of, to recovery from, anaemia [19]. Drug-attributable fall in haematocrit (DAFH) was defined as the difference between pre-treatment and the lowest recorded haematocrit values in the first week after initiation of treatment [21, 22].

### Evaluation of responses to treatment

Response to drug treatment was assessed using a modified version of the World Health Organization *in vivo* clinical classification criteria [26, 27] and other outcome measures, as previously described [7, 13]. Briefly, the outcome measures include asexual parasite positivity on day 1 or 2 after treatment initiation, parasite reduction ratio 1 or 2 days after treatment initiation, parasite clearance time and time to recovery from anaemia.

### Definition of persistent early-appearing anaemia (PEAA)

Haematocrit <30% within 2 days of treatment initiation in a patient who was not anaemic at presentation and its persistence for at least 7 days in the absence of any concomitant illness, was regarded as (PEAA).

### Evaluation of the kinetics of the disposition of the deficit in haematocrit from baseline (DIFHB) in children with PEAA

Patients were evaluated using modified conventional kinetic parameters if they were: non-anaemic at enrolment (haematocrit ≥30% before treatment); developed anaemia within 1–2 days following initiation of treatment and remained anaemic until 1 week after treatment initiation, that is, from days 1 or 2 to day 7, and blood was obtained daily for haematocrit estimation on at least five occasions between days 1–7. Because haematocrit values at presentation were normal in all patients, deficits in haematocrit values from day 1 or 2 after treatment initiation until haematocrit values returned to normal were subtracted from the baseline haematocrit values. Area under the curve of the deficit in haematocrit from baseline (DIHFB) *versus* time (AUC_dihfb_) was estimated by the trapezoidal method, as previously described [20, 23]. Plasma clearance of DIHFB (that is, recovery from the anaemia) was estimated from the equation: CLp_dihfb_ = Haematocrit concentration at time 0 (pre-treatment)/AUC_dihfb_ where CLp_dihfb_ is the volume of blood completely cleared of the deficit in haematocrit/day. *K*_eldihfb_ was estimated from the equation *K*_eldihfb_ = 0.693/*t*_1/2dihfb_, where *K*_eldihfb_ is the elimination rate constant and *t*_1/2dihfb_ is the half-time of DIHFB. For estimation of the volume of distribution of DIHFB, haematocrit values were converted to haemoglobin values by dividing the values by a factor of three, as suggested by Bain and Bates [1]. The volume of distribution of the deficit in haemoglobin (Vd_diHbfb_) was estimated from the equation: Vd_diHbfb_ = CLp_diHbfb_/*K*_eldiHbfb_.

### Statistical analysis

Data were analysed using version 6 of Epi-Info software [8] and the statistical program SPSS for Windows version 20.0 [24]. Proportions were compared by calculating *χ*^2^ using Yates’ correction, Fisher’s exact or Mantel Haenszel tests. Normally distributed, continuous data were compared by Student’s *t*-test and analysis of variance (ANOVA) or by paired *t*-test. Univariate analysis and stepwise multiple logistic regression models were used to test the association between clinical, parasitological or haematological parameters and PEAA, and to evaluate independent predictors of PEAA, respectively. Relationships between two variables that are continuous and normally distributed and those that are discrete and not normally distributed were evaluated by Pearson’s correlation coefficient and Spearman’s correlation coefficient, respectively. Agreement between AnRT and multiples of half-time of DIHFB was assessed by Bland–Altman analysis [2]. Values of *p* < 0.05 were taken to indicate significant differences. Data were double entered serially using patient codes and were only analysed at the end of the study.

### Ethics approval and consent to participate

The study protocol from which the dataset was derived was approved by the National Health Research Ethics Committee, Abuja, Nigeria [NHREC/01/01/2007-22/10/2014]. A written informed consent was obtained from the parents or guardians of the children.

## Results

### Characteristics of patients at enrolment

**Figure 1:**
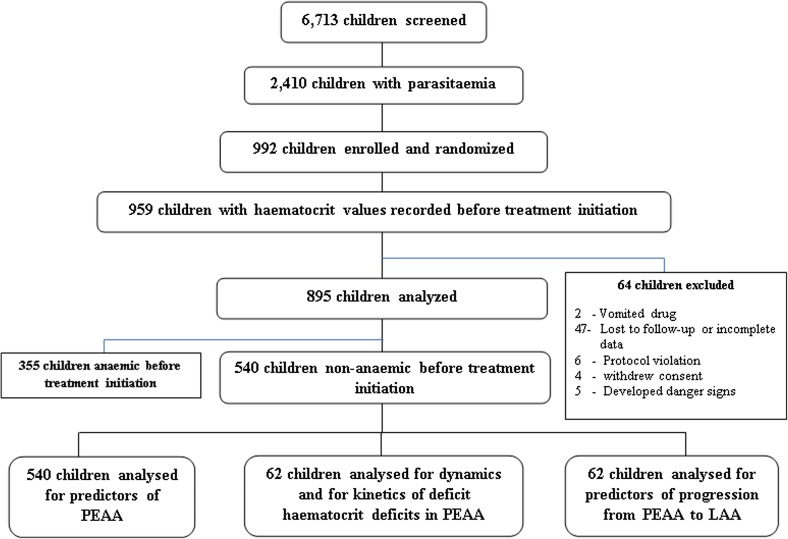
Study flowchart. PEAA, persistent early-appearing anaemia; LAA, late-appearing anaemia.

During the study period, 6713 children with symptoms suggestive of uncomplicated falciparum malaria were screened for *P*. *falciparum*. Parasitaemia was present in 2410 children; 540 children had haematocrit values ≥30% before treatment initiation (Fig. 1). The baseline characteristics of the children with PEAA are summarised in Table 1. Compared to children without PEAA, children with PEAA were significantly younger, weighed significantly less, presented with a history of significantly shorter duration of illness, had a significantly lower haematocrit, and a significantly higher parasite burden.

**Table 1:** Baseline characteristics of the children evaluated.

Parameters	Persistent early-appearing anaemia			*p*-value
	No (*n* = 478)	Yes (*n* = 62)	ALL (*n* = 540)	
Female (%)	223 (46.7)	30 (48.4)	253 (46.9)	0.9
Age				
≤12 months (%)	28 (5.9)	6 (9.7)	34 (6.3)	0.38
>12–36 months (%)	167 (34.9)	33 (53.2)	200 (37)	0.007
>36 months (%)	283 (59.2)	23 (37.1)	306 (56.7)	0.002
Temperature > 37.4 °C (%)	308 (64.4)	40 (65.2)	348 (64.4)	1.0
Haematocrit > 35%	172 (36)	9 (14.5)	181 (33.5)	0.001
Parasitaemia (μL^−1^)				
≤25,000 (%)	271 (56.7)	25 (40.3)	296 (54.8)	0.02
>25,000–50,000 (%)	81 (16.9)	13 (21)	94 (17.4)	0.54
>50,000–100,000 (%)	79 (16.5)	13 (21)	92 (17)	0.49
>100,000 (%)	47 (9.8)	11 (17.7)	58 (10.7)	0.09
Mean value (95% CI)				
Age (month)	41.3 (39.8–42.7)	35.1 (31.1–39.1)	40.6 (39.2–41.9)	0.005
Weight (kg)	13.8 (13.4–14.2)	11.6 (10.8–12.5)	13.6 (13.2–13.9)	<0.0001
Duration of illness (day)	4 (3.6–4.3)	2.8 (2.2–3.5)	3.8 (3.5–4.2)	0.003
Temperature (°C)	37.9 (37.7–38)	37.9 (37.6–38.2)	37.9 (37.8–40)	0.64
Haematocrit (%)	33.8 (33.5–34.1)	32.1 (31.5–32.7)	33.6 (33.3–33.9)	<0.0001
Geometric mean parasitaemia (μL^−1^)	16,175 (14,284–18,316)	28,427 (20,406–39,600)	17,257 (15,350–19,401)	0.003

### Clinical features of children with persistent early-appearing anaemia

#### Frequency and age distribution of children with persistent early-appearing anaemia

**Figure 2:**
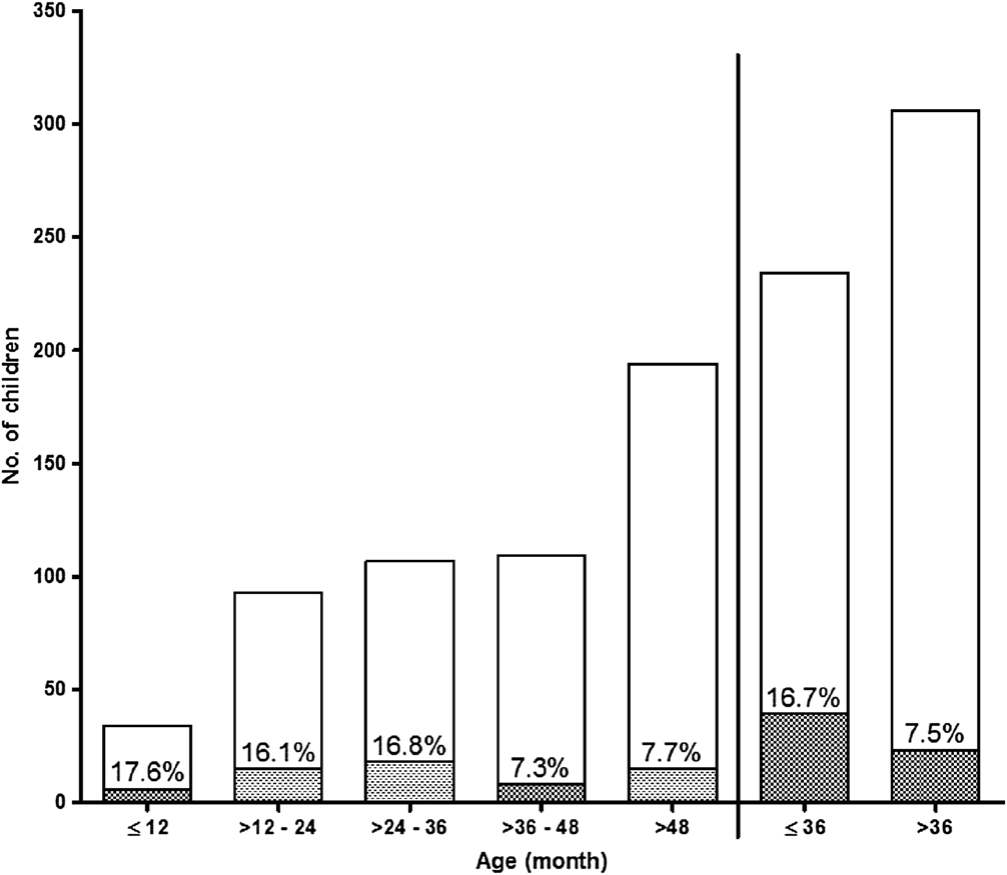
Frequency and age distribution of children with persistent early-appearing anaemia following initiation of artemisinin-based combination treatments of uncomplicated infections.

Sixty two of 540 children with normal haematocrit before treatment initiation had PEAA. PEAA occurred in 10 of 168 (6%), 18 of 176 (10.2%) and 34 of 196 children (17.3%) treated with AA, AL and DHP, respectively – a proportion significantly higher in DHP-treated children (*p* = 0.003). PEAA was significantly more common in children aged 36 months and under compared with those aged >36 months [39 of 234 children (16.7%) *versus* 18 of 306 children (7.5%), respectively; *p* = 0.002; Fig. 2].

### Comparison of symptoms and signs at presentation and during PEAA

**Figure 3:**
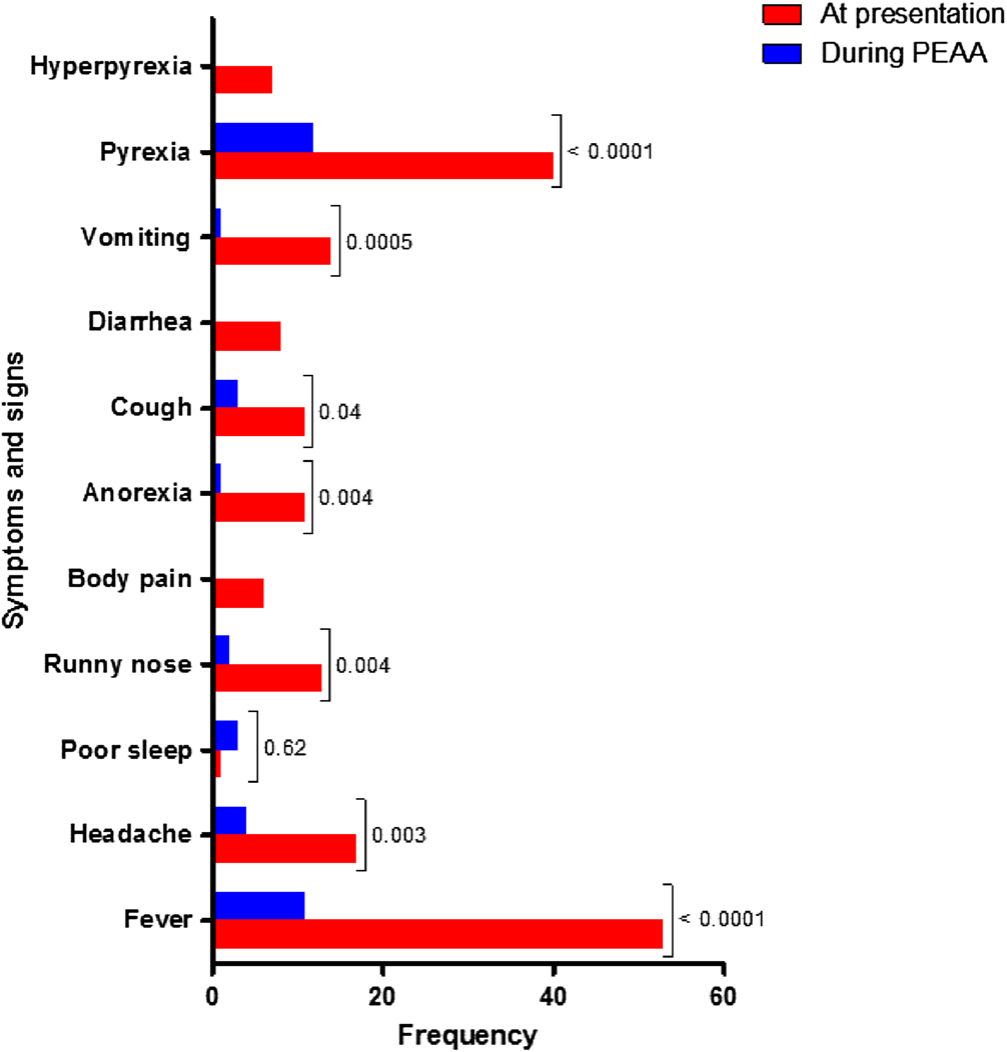
Symptoms and signs before treatment and during persistent early-appearing anaemia in children treated with artesunate-amodiaquine, artemether-lumefantrine or dihydroartemisinin-piperaquine. PEAA, persistent early-appearing anaemia.

The frequency of presenting symptoms and their severity were similar in the 478 children without PEAA and the 62 children who subsequently developed PEAA (data not shown). Sixty one children (98.4%) reported no symptom 1 week after its commencement. In these children, parasitaemia, fever and other symptoms cleared within 3 days of treatment initiation. The frequency of symptoms and signs on presentation and during PEAA are shown in Figure 3. Compared with symptoms at presentation, PEAA was accompanied by significantly fewer symptoms of fever, headache, runny nose, anorexia, cough and vomiting, and signs of pyrexia.

### Factors contributing to persistent early-appearing anaemia following artemisinin-based combination treatments

A duration of illness ≤3 days, enrolment haematocrit <35%, haematocrit 1 day after treatment initiation <25%, DAFH ≥6%, and treatment with DHP independently predicted PEAA (Table 2).

**Table 2:** Predictors of persistent early-appearing anaemia in acutely malarious <5-year-old children following initiation of artemisinin-based combination treatments.

Variable	Total no.	No. with PEAA	OR (95% CI)	*p* value	AOR (95% CI)	*p* value
Gender						
Female	253	30	1			
Male	287	32	0.9 (0.5–1.6)	0.9	–	–
Age (month)						
>36	306	23	1		1	
≤36	234	39	2.5 (1.4–4.3)	0.002	1.8 (0.5–4.3)	0.18
Duration of illness (day)						
>3	185	17	1		1	
≤3	180	30	2.0 (1.0–3.7)	0.048	2.9 (1.1–7.4)	0.03
History of fever at presentation						
Absent	90	9	1			
Present	450	53	1.2 (0.6–2.5)	0.73	–	–
Temperature at presentation (^°^C)						
≤37.4	192	22	1			
>37.4	348	40	1.0 (0.6–1.7)	1.0	–	–
History of fever on day 1						
Absent	446	55	1			
Present	94	7	0.6 (0.3–1.3)	0.24	–	–
Temperature on day 1 (°C)						
≤37.4	480	55	1			
>37.4	60	7	1.0 (0.4–2.4)	1.0	–	–
Enrolment haematocrit (%)						
≥35	181	9	1		1	
<35	359	53	3.3 (1.6–6.9)	0.001	4.6 (1.6–13.1)	0.005
Haematocrit on day 1 (%)						
≥25	492	42	1		1	
<25	48	20	7.7 (4–14.7)	<0.0001	3.5 (1.1–11.4)	0.03
DAFH (%)						
<6	333	17	1		1	
≥6	207	45	5.2 (2.9–9.3)	<0.0001	5.7 (2.1–15.5)	0.001
Enrolment parasitaemia (/μL)						
≤75,000	454	45	1		1	
>75,000	86	17	2.2 (1.2–4.1)	0.02	1.3 (0.4–4.1)	0.65
Asexual parasitaemia on day 1						
Absent	209	24	1			
Present	331	38	1.0 (0.6–1.7)	1.0	–	–
Asexual parasitaemia on day 2						
Absent	422	51	1			
Present	118	11	0.7 (0.4–1.5)	0.5	–	–
Parasite clearance time (day)						
≤2	418	51	1			
>2	122	11	0.7 (0.4–1.4)	0.42	–	–
Parasite reduction ratio on day 1						
≤25,000	453	44	1			
>25,000	87	18	1.4 (0.8–2.6)	0.31	–	–
Parasite reduction ratio on day 2						
≤25,000	339	31	1		1	
>25,000	201	31	1.8 (1.1–3.1)	0.04	1.2 (0.5–3.1)	0.7
Drug treatment						
AA	168	10	1		1	
AL	176	18	1.8 (0.8–4.0)	0.22	–	–
DHP	196	34	3.3 (1.6–6.9)	0.002	4.3 (1.6–11.5)	0.004

DAFH, drug attributable fall in haematocrit; DHP, dihydroartemisinin-piperaquine; AL, artemether-lumefantrine; AA, artesunate-amodiaquine; OR, odds ratio; AOR, adjusted odds ratio; PEAA, persistent early appearing anaemia.

### Recovery from persistent early-appearing anaemia

On day 1 following treatment initiation, PEAA was mild or moderate in 43 and 3 children, respectively. On day 2, it was mild or moderate in 15 children and 1 child, respectively. On day 7, it was mild or moderate in 60 and 2 children, respectively. During a follow-up period of 42 days, 7 children did not recover from their PEAA. The proportions of children who did not recover from their PEAA were similar with all three treatments [1 of 10 children (10%) *versus* 4 of 18 children (22.2%) *versus* 2 of 34 children (5.9%) treated with AA, AL or DHP, respectively; *p* = 0.21]. Overall, mean of time to recovery from PEAA was 17.4 days (95% CI 15.5–19.4, *n* = 55) and it was similar with all three treatments [15.9 days (95% CI 13–18.7, *n* = 9) *versus* 20.7 days (95% CI 15.5–26, *n* = 14) *versus* 16.4 days (95% CI 14–18.9, *n* = 32) in AA-, AL- and DHP-treated children, respectively; *p* = 0.14].

#### Other dynamics of recovery from PEAA

On day 7, overall deficit from pre-treatment haematocrit was 5.7% (95% CI 4.8–6.6) and it was similar with all three treatments [5.4% (95% CI 3–7.2, *n* = 10) *versus* 5.4% (95% CI 4.2–6.6, *n* = 18) *versus* 6% (95% CI 4.8–7.5, *n* = 34), in AA-, AL- and DHP-treated children, respectively; *p* = 0.77].

- Time to 50% reduction of DIHFB

Overall, mean time to 50% reduction in DIHFB (*T*_50dihfb_) was 12.1 days (95% CI 9.6–14.2, *n* = 59) and it was similar with all three treatments [11.4 days (95% CI 8.2–14.6, *n* = 10) *versus* 15.4 days (95% CI 9.9–20.9, *n* = 17) *versus* 10.5 days (95% CI 8.6–12.5, *n* = 32), in AA-, AL- and DHP-treated children, respectively, *p* = 0.09].

- Time to 90% reduction of DIHFB

Overall, mean time to 90% reduction in DIHFB (*T*_90dihfb_) was 16.7 days (95% CI 14.9–18.4, *n* = 53) and it was significantly longer in AL-treated compared with AA- or DHP-treated children [20.5 days (95% CI 15.8–25.2, *n* = 16) *versus* 15.4 days (95% CI 12.8–18, *n* = 10) *versus* 14.9 days (95% CI 13.2–16.6, *n* = 27), respectively, *p* = 0.01]. Time to 90% reduction in haematocrit deficit was similar in AA- and DHP-treated children (*p* = 0.98).

#### Kinetics of the disposition of the deficit in haematocrit from baseline

**Figure 4:**
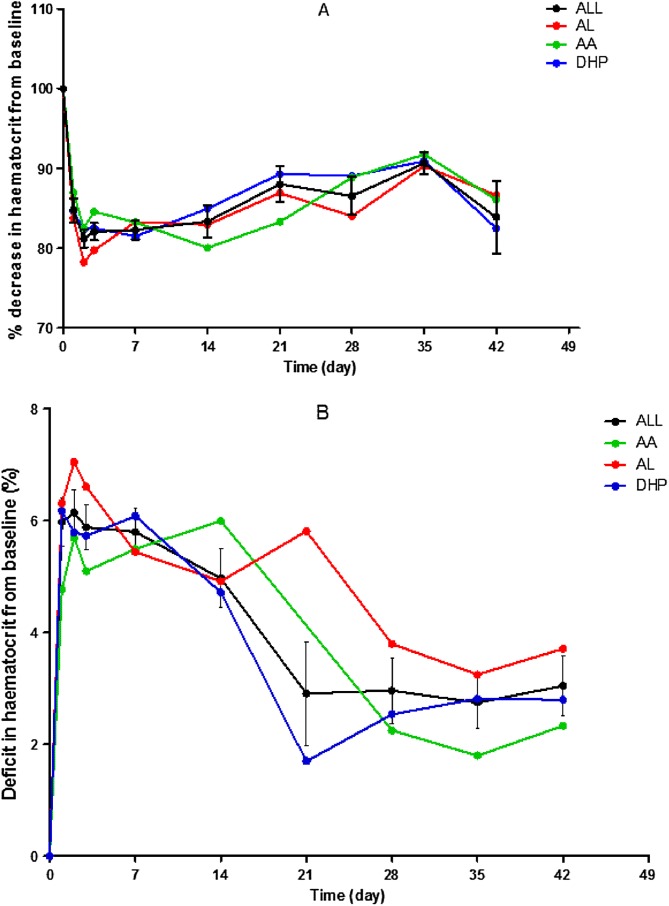
Time-course of percentage decrease (A) and of deficit in haematocrit (B) from baseline in all children (black line) and in those treated with artesunate-amodiaquine (green line), artemether-lumefantrine (red line) or dihydroartemisinin-piperaquine (blue line). Values are means and standard errors of mean; measures of dispersion have been included only in black plots for clarity.

The time-course of percentage decrease and of the disposition of DIHFB are shown in Figure 4. Mean percentage decrease in DIHFB was 18.4% (range 3–40, *n* = 53), 18.8% (range 3.3–42.9, *n* = 62), 18.9% (range 3.7–36.8, *n* = 62), 17.7% (range 3.7–46.9, *n* = 62), 16.6% (range 3.2–38.7, *n* = 25), 11.9% (range 3.3–13.4, *n* = 10), 13.4% (range 3.3–19.4, *n* = 9), 9.3% (range 6.7–13.9, *n* = 9) and 16.1% (range 10–25, *n* = 8) on days 1, 2, 3, 7, 14, 21, 28, 35, and 42, respectively (Fig. 4A). Maximum deficit occurred between 2 and 3 days after treatment initiation and it declined gradually thereafter (Fig. 4B). Overall, mean AUC_dihfb_ was 87.4% day (95% CI 70.5–104.1, *n* = 62) and it was similar for all three treatments [72.6% day (95% CI 34.1–111.1, *n* = 10) *versus* 104.3% day (95% CI 68.8–139.9, *n* = 18) *versus* 82.7% day (95% CI 59.6–105.9, *n* = 34), in AA-, AL- and DHP-treated children, respectively, *p* = 0.4].

**Figure 5:**
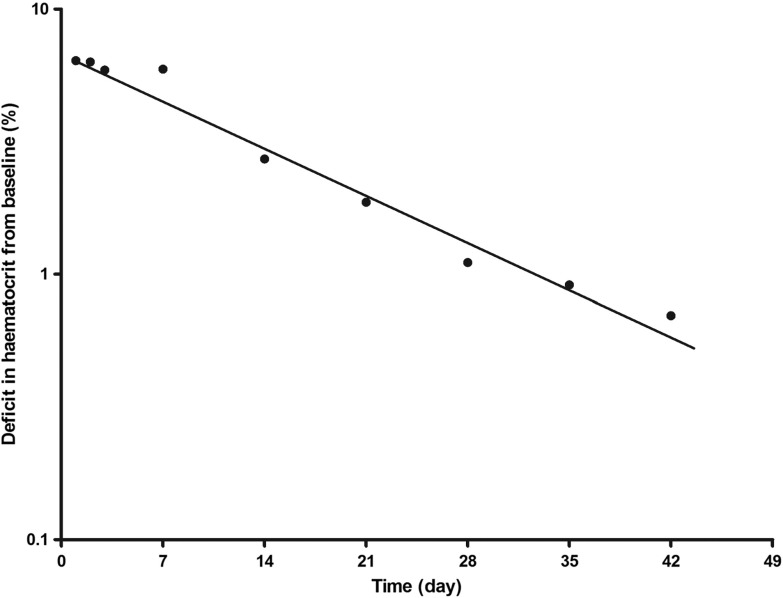
Semilogarithmic plot of deficit in haematocrit from baseline *versus* time in all children who developed persistent early-appearing anaemia following initiation of treatment with artesunate-amodiaquine, artemether-lumefantrine or dihydroartemisinin-piperaquine.

Declines in DIHFB were monoexponential with overall mean estimated half-time of 3.9 days (95% CI 2.5–5.1, *n* = 62, Fig. 5). Mean estimated half-time was similar with all three treatments [2.2 days (95% CI 1.5–2.9, *n* = 10) *versus* 5.1 days (95% CI 1.8–8.4, *n* = 18) *versus* 3.7 days (95% CI 2.1–5.3, *n* = 34) in AA-, AL- and DHP-treated children, respectively; *p* = 0.45]. The other kinetic parameters of the disposition of DIHFB are summarised in Table 3. These parameters are similar with all three treatments (Table 3).

**Table 3:** Kinetic parameters of the disposition of the deficit in haematocrit from baseline in children with persistent early-appearing anaemia following initiation of treatment.

Parameters	Artesunate-amodiaquine	Artemether-lumefantrine	Dihydroartemisinin-piperaquine	ALL	*p* value
	(*n* = 10)	(*n* = 18)	(*n* = 34)	(*n* = 62)	
Cmax_dihfb_ (%)					
Mean (95% CI)	6.2 (3.8–8.6)	8.4 (6.8–10.1)	7.5 (6.3–8.7)	7.6 (6.7–8.4)	0.25
Tmax_dihfb_ (day)					
Mean (95% CI)	3.3 (1.5–5.1)	2.7 (1.7–3.8)	3.8 (2.6–5.1)	3.4 (2.6–4.1)	0.46
AUC_dihfb_ (%.day)					
Mean (95% CI)	72.6 (34.1–111.1)	104.3 (68.8–139.9)	82 (59.6–105.9)	87.4 (70.6–104.1)	0.4
*t*_1/2dihfb_ (day)					
Mean (95% CI)	2.2 (1.5–2.9)	5.1 (1.8–8.4)	3.7 (2.1–5.3)	3.9 (2.6–5.1)	0.29
*K*_eldihfb_ (day^−1^)					
Mean (95% CI)	0.3 (0.3–0.4)	0.3 (0.2–0.3)	0.3 (0.2–0.4)	0.3 (0.3 -0.4)	0.26
CLp_dihfb_ (%/day)					
Mean (95% CI)	0.7 (0.2–1.2)	0.4 (0.3–0.4)	0.6 (0.4–0.8)	0.6 (0.5–0.7)	0.13
Vd_diHbfb_ (L/Kg)					
Median (Range)	0.16 (0.04–0.95)	0.17 (0.06–0.43)	0.19 (0.06–0.62)	0.17 (0.04–0.95)	0.8

Cmax_dihfb_, maximum deficit in haematocrit from baseline; Tmax_dihfb_, time to reach maximum deficit in haematocrit from baseline; AUC_dihfb_, area under the curve of deficit in haematocrit from baseline *versus* time; *t*_1/2def_, elimination half-time of deficit in haematocrit from baseline; *K*_eldihfb_, elimination rate constant of deficit in haematocrit from baseline; CLp_dihfb_, volume of blood completely cleared of the deficit in haematocrit from baseline; Vd_diHbfb_, volume of distribution of the deficit in haemoglobin from baseline; L, litre; ALL, all children.

#### Relationship between time to reduction of deficit in haematocrit from baseline and half-time of haematocrit deficit

**Figure 6:**
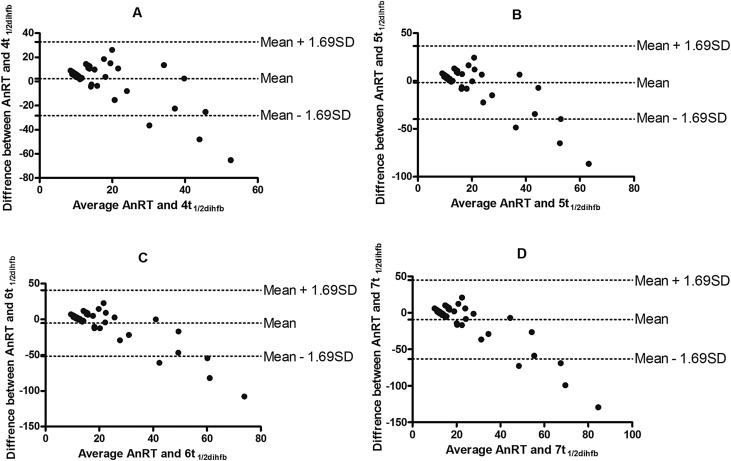
Bland-Altman plots of anaemia recovery time (AnRT) and multiples [4 (A), 5 (B), 6 (C) and 7 (D)] of half-time of deficit in haematocrit from baseline in children with persistent early-appearing anaemia. Biases were 2.23, −1.57, −5.37 and −9.17 for plots A, B, C and D; *p* = 0.29, 0.55, 0.09 and 0.02, respectively. The mean values ± 1.96 standard deviation (*SD*) of the differences are shown. dihfb, deficit in haematocrit from baseline.

There was a significant positive correlation between the following parameters: half-time of DIHFB and time to 50% reduction in DIHFB (*r* = 0.54, *p* < 0.0001, *n* = 57); half-time of DIHFB and time to 90% reduction in DIHFB (*r* = 0.54, *p* < 0.0001, *n* = 53); half-time of DIHFB and time to recovery from PEAA (*r* = 0.37, *p* = 0.005, *n* = 55); and 5 multiples of half-time of DIHFB and AnRT (*r* = 0.33, *p* = 0.01, *n* = 55). The mean ratio of AnRT to mean of half-time of DIHFB (*t*_1/2dihfb_) was 7.1 (95% CI 6.1–8.1). In Bland-Altman analyses, there were narrow limits of agreement between AnRT and 4, 5 or 6 multiples of half-time of DIHFB (Fig. 6). The limits of agreement were −28.4 to 32.8, −39.8 to 36.6 and −51.4 to 40.6 at 4, 5 or 6 multiples of half-time of haematocrit deficit, respectively. The bias at the multiples of 4, 5 or 6 half-time was statistically insignificant (*p* = 0.29, 0.55 or 0.1, respectively). However, there was statistically significant bias at multiples of 7 half-time of DIHFB (*p* = 0.02, Fig. 6).

### Relationship between persistent early-appearing anaemia and late-appearing anaemia

Ten of the 55 children (18%) who recovered from their PEAA after day 14 subsequently progressed to asymptomatic LAA (Table 4). The mean of time from recovery from PEAA to onset of LAA was 12 days. Proportions of children who recovered from their PEAA and subsequently progressed to LAA were significantly lower in children treated with DHP compared with AA- or AL- treated children (2 of 34 children (5.9%) *versus* 4 of 10 children (40%) *versus* 4 of 18 children (22.2%), respectively; *p* = 0.02). Nadir haematocrit during PEAA was similar with nadir haematocrit during LAA (Table 4). Following initiation of treatment, the mean of haematocrit, at all times in these children was <30%. Six of the 10 children who developed LAA did not recover from their LAA on day 42 of follow-up.

**Table 4:** Features of children who recovered from persistent early-appearing anaemia and who subsequently progressed to late-appearing anaemia.

Patient (gender, age)	Parasitaemia (μL^−1^)	Pre-treatment HCT (%)	Antimalarial treatment	PRRD2	PCT (days)	Nadir HCT at PEAA (%)b,c	Anaemia recovery time (day)	Time to LAA (day)^d^	Nadir HCT at LAA (%)b,c	HCT on day 42 (%)	Time between recovery and LAA (day)
AD57 (M, 24m)	53,678	36	DHP	53,678	2	25	33	42	27	27	7
AD67 (F, 38m)	23,670	30	AA	23,670	1	26	13	35	26	30	21
IM/E/47 (F, 59m)	4846	30	AA	4846	2	26	20	35	28	28	14
IM/E/50 (F, 14m)	151,733	30	AA	151,733	2	25	20	42	25	25	21
IM/E/66 (F, 59m)	27,965	30	AL	27,965	1	26	13	21	28	28	7
KN/EN/90 (F, 24m)	30,166	30	DHP	30,166	1	23	13	35	29	38	21
KW10 (F, 42m)	120,050	34	AL	5002	3	23	20	28	29	32	7
SK18 (M, 16m)	4842	33	AL	4842	2	26	13	35	25	26	21
SK31 (F, 27m)	8538	31	AL	94.87	3	20	13	28	20	25	14
SK46 (F, 19m)	2004	31	AA	11.93	3	24	19	28	23	–	7
**Mean (95% CI)**	**19,473** a	**31.5 (30–33)**		**5413** a	**1.8 (1.3–2.5)**	**24.4 (23–25.8)**	**16.9 (13.5–21.1)**	**32.3 (27.7–37.6)**	**26 (23.9–28)**	**27.8 (25.6–32)**	**12 (8.6–18.1)**

m, month; AA, artesunate-amodiaquine; AL, artemether-lumefantrine; DHP, dihydroartemisinin-piperaquine; PCT, parasite clearance time; HCT, haematocrit; CI, confidence interval; PEAA, persistent early-appearing anaemia; LAA, late-appearing anaemia.

a Geometric mean.

b No significant difference between nadir haematocrit during PEAA and LAA (*p* = 0.08).

c Virtually all patients were asymptomatic during PEAA and LAA.

d Time from commencement of treatment to occurrence of LAA.

### Factors contributing to progression from persistent early-appearing anaemia to late-appearing anaemia following artemisinin-based combination treatments

In a univariate analysis, female gender [odds ratio (OR) = 5.5 (95% CI 1.1–28.2)] and treatment with AA [OR = 10.7 (95% CI 1.6–71.9)] were significantly associated with progression from PEAA to LAA (*p* = 0.04 and 0.02, respectively). In a multivariate analysis, none of the associated factors independently predicted progression from PEAA to LAA [adjusted odds ratio (AOR) = 4.1 (95% CI 0.4–45.3, *p* = 0.25) and AOR = 6.7 (95% CI 0.9–50.5, *p* = 0.07) for female gender and treatment with AA, respectively].

### Adverse events

Sixty one of 540 children (11%) reported at least one adverse event in the 1 week following initiation of treatment. The proportions of children reporting adverse events were similar in those with [7 of 62 children (11%)] and those without [54 of 478 children (9%)] PEAA. Reported adverse events were indistinguishable from those reported at presentation and consisted mainly of headache, nausea, cough or fever.

## Discussion

In this study of children with uncomplicated falciparum malaria conducted over a period of 2 years, 62 of 540 children (11.5%) developed PEAA following treatment with AA, AL or DHP. Virtually all of the children with PEAA were asymptomatic. The asymptomatic nature of the anaemia made it difficult, in the absence of haematocrit estimation, to make a diagnosis of PEAA. In addition, without intervention, many of the children (89%) recovered from PEAA, but about 18% of those who recovered progressed to late-appearing anaemia.

Amongst the factors contributing to PEAA were young age, heavy parasite burden, and rapid reduction of heavy parasitaemias – the hallmark of artemisinins in artemisinin-sensitive infections. Collectively, these factors indicate that the interplay of host, parasite and drug factors made PEAA 2–6 times more likely than in those without these attributes [21]. Two points may possibly help explain the persistence of anaemia for one or more weeks. First is continuing low grade haemolysis following the initial 18% decrease in haematocrit from baseline (Fig. 4), and second, slow recovery from the initial considerable falls in haematocrit following initiation of treatment. Thus, it is likely PEAA in uncomplicated falciparum malaria in children may be analogous to “persistent type post-treatment anaemia” following intravenous artesunate treatment of severe malaria in immunologically naïve adults [9]. In keeping with this analogy is the similar mean drop (18% *versus* 16%) in baseline haematocrit three or more days after initiation of treatment in both situations (Fig. 4). Although DHP treatment predicted PEAA, PEAA was not related to the dose of dihydroartemisinin or piperaquine (data not shown). We have no explanation for this observation.

Although PEAA resulted in a mean of approximately 6% deficit in haematocrit (17.7% drop from baseline) by 1 week after initiation of treatment, recovery of the deficit occurred in 89% of the children by 17–18 days after treatment initiation. Kinetically, recovery of the deficit was a first-order process with an estimated mean half-time of 4 days. If after 4 or 5 half-times 94 or 97%, respectively of a first-order process would have been completed [17], it follows that approximately 16–20 days would be required for recovery from ACTs-related PEAA. Thus, there is correlation and agreement between recovery from PEAA determined dynamically and kinetically in the same individual in the cohort of children we evaluated. The insignificant bias on Bland-Altman analyses, between AnRT and 4 or 5 multiples of half-times of DIHFB (Fig. 6) is in keeping with this conclusion. The agreement also indicates AnRT or 4, 5 or 6 multiples of half-times of DIHFB can be used interchangeably in the same patient.

The other outcomes of asymptomatic PEAA included non-recovery from PEAA in 11% of the children, and after recovery, progression to asymptomatic LAA in another 18% of the children. Taken together, these outcomes suggest ACTs predisposed 27% of young non-anaemic children with malaria before treatment initiation to a relatively prolonged period of anaemia following initiation of treatment. ACTs may also predispose these children to chronic anaemia if the malaria infections are frequent and the time available for complete recovery from anaemia in between infections is insufficient [19]. Strategies to reduce the likelihood of ACT-related PEAA and its consequences are urgently required in young children and should include early diagnosis and prevention. Estimation of haematocrit or haemoglobin before treatment initiation, on the last day of direct observed therapy (DOT), and at 2 weeks after initiation of treatment should aid in the early diagnosis of PEAA.

It is likely some differences may exist in the time-course of anaemia following treatments with the three ACTs. For example, DHP treatment predicted PEAA but recovery from PEAA was not unduly prolonged. Children treated with DHP also had significantly reduced propensity of their recovered PEAA for subsequent progression to LAA when compared to those treated with AA or AL (Table 4). On the other hand, both AA- and AL-treated children appeared to have significantly lesser propensity to develop PEAA, perhaps due to lag-times for conversion of artesunate or artemether to dihydroartemisinin but the children have significantly higher propensity of their recovered PEAA to progress to LAA (Table 4). An important feature of AL-related anaemia is slow recovery from PEAA, which, in part, is evidenced by the significantly longer time to 90% reduction in DIHFB.

It is unclear why female gender and treatment with AA were significantly associated with progression to LAA of children who recovered from their PEAA. It is also unclear the mechanisms for progression of PEAA to LAA. Rapid reduction of parasitaemia, evidenced by a PRRD2 value >25,000 being significantly related to PEAA (Table 2), should result in generation, by the artemisinin components of ACTs, of a significant number of pitted red blood cells that may be destroyed 7–21 days after treatment initiation to produce LAA [9]. Quantification of once-infected red blood cells in those who progressed and in those who did not progress from PEAA to LAA may aid both in identification of the causes or mechanisms and the risk of progression. It is also possible that early destruction of once-infected red blood cells may contribute to producing PEAA from EAA.

The criterion for onset of anaemia in patients with PEAA within 2 days after initiation of treatment took into account an intra-erythrocytic cycle of approximately 48 h, a period of exclusive treatment with study drugs and of maximum exposure to these drugs, and an end of time of complete or almost complete clearance of parasitaemia in sensitive infections. Collectively, these allow for evaluation of the contribution of host, parasite and drug factors to producing EAA and its persistence for one or more weeks after treatment initiation (see above). In this context, it is likely that, in the setting of a parasitaemia <200,000/μL, age <5 years, haematocrit >30% before treatment initiation, a >15% decrease in baseline (pre-treatment) haematocrit on day 2 and/or 3 with or without available raised plasma lactate dehydrogenase and/or low haptoglobin, a case definition of PEAA or ACT-related “persistent post-treatment anaemia syndrome” can be made in these children in the settings of intense transmission.

Use of the DIHFB as a method for kinetic analyses of recovery from PEAA allowed for demonstration of the monoexponential declines from peak deficit in haematocrit, the hallmark of a first-order process [17]. It also confirms recovery from PEAA is a first-order process. The method is similar to that for estimation of half-life of a drug in a one-compartment pharmacokinetic model following a constant rate intravenous infusion in which drug concentrations prior to attainment of steady state or plateau are subtracted from steady state or plateau concentration and plotted against time semilogarithmically [17]. In this model, declines in drug concentrations are monoexponential [17].

There are limitations of the present study. First is non-determination of whether PEAA is haemolytic or non-haemolytic. Measurement of plasma lactate dehydrogenase and/or haptoglobin would have assisted with this determination. Second is non-quantification of once-infected erythrocytes. Quantification would have permitted evaluation of their contribution to PEAA and its progression to LAA. Third is non-quantification of reticulocytes. Quantification would have allowed evaluation of the contribution of reticulocytopaenia to PEAA. In animals, artemisinins cause reticulocytopaenia by suppressing erythroblasts [5]. Forth is the non-measurement of plasma dihydroartemisinin levels and their relationship to PEAA, since treatment with DHP predicted PEAA and both artesunate and artemether are converted to dihydroartemisinin. Finally, in the estimation of Vd_diHbfb_, we did not measure haemoglobin since the conversion of haematocrit to haemoglobin by a factor of three as suggested by Bain and Bates [1] may not hold true especially when haemoglobin values are low [4, 15].

In conclusion, asymptomatic PEAA is common in young non-anaemic children with malaria following initiation of ACTs. Its occurrence, or progression to LAA, may have implications for case and community management of anaemia and for anaemia control efforts in sub-Saharan Africa where ACTs have become first-line antimalarials. Recovery from PEAA is a first-order process.

## Data Availability

The dataset supporting the findings of this article is available from the corresponding author upon request.

## Notes

**Cite this article as**: Akano K, Fatunmbi B, Ntadom G, Ayede AI, Aderoyeje T, Bakre A, Alebiosu OT, Akpoborie O, Okafor C, Gbotosho GO, Folarin OA, Ebenebe JC, Ambe J, Wammanda R, Jiya N, Finomo F, Emechebe G, Mokuolu O, Agomo C, Oguche S, Happi C & Sowunmi A. 2019. Clinical illness and outcomes in Nigerian children with persistent early-appearing anaemia following initiation of artemisinin-based combination treatments of uncomplicated falciparum malaria. Parasite **26**, 56.
